# Reforming early intervention for premature infants: insights into integrated nursing and medical care in Western China

**DOI:** 10.3389/fped.2024.1469757

**Published:** 2024-12-24

**Authors:** Meicen Zhou, Xin Lin, Huan Luo, Haiting Liu, Shaopu Wang, Hua Wang, Dezhi Mu

**Affiliations:** ^1^ Department of Pediatrics, West China Second University Hospital, Sichuan University, Chengdu, Sichuan, China; ^2^ Key Laboratory of Birth Defects and Related Diseases of Women and Children, Ministry of Education, Sichuan University, Chengdu, Sichuan, China; ^3^ Department of Neonatology, Fujian Maternity and Child Health Hospital/College of Clinical Medicine for Obstetrics & Gynecology and Pediatrics, Fujian Medical University, Fuzhou, Fujian, China; ^4^ Department of Pediatrics, Wuhou District People’s Hospital, Chengdu, Sichuan, China

**Keywords:** cerebral intraventricular hemorrhage, delivery rooms, intensive care, nomograms, preterm infants

## Abstract

**Background:**

Premature births has imposed substantial burdens on medical resources. Consequently, a specialized team was established and a model focused on early intervention, namely the Delivery Room Intensive Care Unit (DICU) emphasizing “care, support, and treatment” was introduced and its impact on the morbidity and mortality outcomes of newborns was assessed. Additionally, we aimed to develop a nomogram model for predicting the risk of intraventricular hemorrhage (IVH) in preterm infants.

**Methods:**

A retrospective study involving 2,788 infants was conducted to compare the characteristics and outcomes of infants admitted following the transition from the previous “neonatal intensive care unit (NICU)-centered” approach to the current early “care, support, and treatment” model. Clinical and laboratory data were recorded from birth until their discharge. The primary outcome was IVH, with additional evaluation of mortality and morbidities related to the neurological, respiratory, circulatory, and digestive systems.

**Results:**

The DICU approach significantly declined the incidence of IVH [OR: 0.16, 95% CrI (0.11,0.23)], hypothermia [OR: 0.33, 95% CrI (0.21,0.50)], apnea [OR: 0.60, 95% CrI (0.47,0.75)], perinatal respiratory diseases [OR: 0.63, 95% CrI (0.52,0.75)] and metabolic acidosis [OR: 0.24, 95% CrI (0.16,0.34)]. Five predictors were selected: DICU exposure, gestational age, birth weight, ventilation mode within seven days, and ibuprofen use (d). The model built by these predictors displayed good prediction ability with the area under the ROC curve of 0.793 in the training set and 0.803 in the validation set.

**Conclusions:**

The standardized DICU model had significantly reduced the incidences of morbidities. The risk nomogram is useful for prediction of IVH risk in eligible infants, with a high accuracy, sensitivity, consistency, and practicability. This study emphasizes the shift in early intervention concepts and team collaboration sets “neonatologists, neonatal nurse practitioners, and respiratory therapists”, which advocates for standardized decision-making for treatment from the delivery room to improve the success rate of resuscitation and enhance the prognosis of these infants.

## Introduction

Preterm birth continues to be a significant public health issue in China, the overall preterm birth rate in China gradually increased, with a notable rise from 5.9% in 2012 to 6.4% in 2018 (1). The implementation of China's universal two-child policy led to an increase in pregnancies among women of advanced maternal age, a major risk factor for preterm deliveries. Geng et al. reported that while the proportion of preterm multiple births significantly increased after the policy, the proportion of live multiple births among all live births significantly declined (2).The mortality rate of preterm infants is estimated to be 12 times higher than that of full-term infants (3). In addition, the preterm infants continue to impose a disproportionately heavy burden of neonatal morbidities and long-term developmental disability in China (4). Therefore, it is imperative and timely to take essential actions to reduce the incidence of preterm deliveries and improve the life quality of preterm infants later in life.

Care practices during the first hour, often referred to as the “Golden Hour”, play a pivotal role in determining the short- and long-term outcomes of preterm infants, particularly those with very low birth weight (5). Evidence-based interventions during this critical period have demonstrated significant benefits in reducing the incidence of neonatal complications such as hypothermia, Intraventricular hemorrhage (IVH) (6), bronchopulmonary dysplasia (7), and retinopathy of prematurity (ROP) (5, 8). Despite global advancements in neonatal care, the application of these practices remains challenging in many regions, including China.

China has made notable progress in the care of critically ill preterm infants through the establishment of neonatal intensive care units (NICUs). For pregnant women at high risk of complications during childbirth, concentrating them in tertiary neonatal medical institutions has been proven to be crucial for reducing neonatal morbidity and mortality rates (9). However, the traditional NICU-centered model has inherent limitations. Transfer delays between delivery rooms and NICUs, coupled with coordination challenges during transport, often lead to suboptimal stabilization during the critical Golden Hour. Additionally, the lack of immediate specialized care at delivery further limits the timely initiation of evidence-based interventions. Prolonged transport times and various critical changes can also impact the effectiveness of treatment (10). These barriers necessitate the exploration of the forward-moving model, which integrates early intervention directly in the delivery room to address these shortcomings.

At West China Second University Hospital, a national center for critical maternal and pediatric care, we have witnessed a growing influx of obstetric and pediatric patients from surrounding regions. In response, we developed a novel neonatal resuscitation model termed the “Delivery Intensive Care Unit” (DICU). This model integrates neonatologists, neonatal nurse practitioners, and respiratory therapists into the resuscitation process for preterm infants born at gestational ages (GA) < 34 weeks and high-risk infants ≥34 weeks. By initiating advanced resuscitation measures in the delivery room or operating room, the DICU aims to provide early respiratory and circulatory support, reducing the risk of severe IVH and other complications, while facilitating seamless NICU transitions.

Intraventricular hemorrhage (IVH) in preterm infants is a significant contributor to neonatal morbidity and mortality, with long-term implications for neurological development (11). Severe IVH, particularly in very low birth weight and extremely low birth weight infants, is associated with a higher risk of cerebral palsy, cognitive impairments, and other developmental disabilities (12–14). These long-term complications substantially affect the quality of life for affected individuals and place a considerable burden on families. Additionally, the emotional toll on parents, coupled with the uncertainty regarding the child's health and future outcomes, can be profound. Beyond the emotional and psychological impact, the management of infants with IVH and its associated complications imposes significant financial burdens on both healthcare systems and society, including long-term medical care, rehabilitation, and special education services (15). Given the preventable nature of IVH through timely and appropriate neonatal interventions (16, 17), reducing its incidence represents a critical goal in neonatal care. While other complications are also critical outcomes.

In this study, we aimed to examine the impact of DICU exposure on neonatal resuscitation practices, whether this management model can effectively optimize care practices from delivery to NICU and ultimately reduce neonatal morbidity and morbidities. Additionally, we explored the effectiveness of this model across different GA groups. To predict IVH outcomes, we developed and validated a nomogram model incorporating DICU exposure, providing a robust tool for assessing its prognostic value. Our findings aim to inform evidence-based strategies for improving preterm infant outcomes within resource-constrained settings.

## Materials and methods

We performed a retrospective study on preterm infants at Sichuan University West China Second University Hospital. Infants who were transferred from the DR to the neonatal department for treatment and discharged from August 2014 to June 2019 were included. Data were collected from electronic medical records. And this study is reported according to the STROBE reporting checklist (Supplementary Table S9).

The exclusion criteria were preterm infants with the following conditions: ① Infants whose total hospitalization was less than 24 h after admission, particularly those where life-sustaining treatment was withdrawn due to poor prognosis and family decision. ② Maternal or infant clinical data were incomplete.

### Comparison groups

The study population data was categorized as the DICU group and non-DICU group, which was recorded in the electronic medical record system. Specific allocation of personnel for two groups was as follows:

Non-DICU group: a team comprised of rotating pediatricians, DR nurses, and midwives before December 2016. The group was responsible for overseeing the entire process from admission to transfer to the NICU for premature infants.

DICU group: Since January 2017, a multidisciplinary neonatal transfer team consisted of neonatologists, neonatal nurse practitioner, and respiratory therapists has been established. Upon receiving notification, the team promptly initiates an assessment of the infant's basic characteristics through remote telephone consultations, and conducts a comprehensive reassessment and observation upon the infant's arrival at the designated hospital. Throughout the transfer process, individualized safety measures are strictly implemented based on potential risk factors to ensure the well-being of each infant. The team is actively involved in overseeing the entire process of transferring premature infants (<34 weeks) and high-risk premature infants from the delivery room (DR) to the neonatal intensive care unit (NICU).

Furthermore, eligible preterm infants were separated into two groups (non-IVH vs. IVH), since then we randomly divided the infants into a training set and a validation set for internal validation conformed to the theoretical ratio of 7:3. The training set was used to find the independent predictors for IVH, and these predictors were then used to build the regression model. The validation set was used to verify the accuracy of the prediction model.

### Exposure

**Figure 1:**
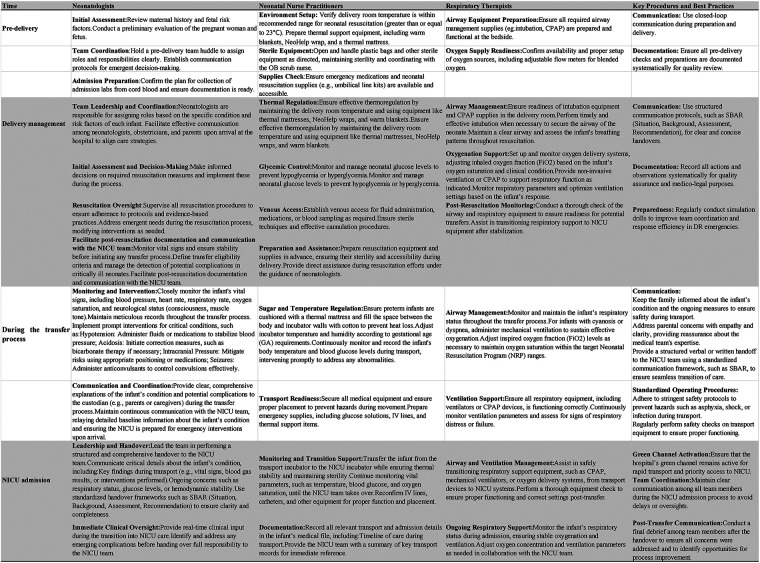
Standardized care practices for DICU transfer process.

The specific standardized care practices during the transfer process in the DICU group were designed based on the roles of different team members, including neonatologists, neonatal nurse practitioners, and respiratory therapists. We provided a detailed framework outlining the responsibilities and procedures associated with each role (Figure 1), aimed at enhancing the consistency and reproducibility of the intervention.

### Outcomes

The outcomes of interest were neonatal morbidities:

The primary outcomes were IVH. IVH was defined as any grade of IVH seen during cranial ultrasound within the first 28 days of an infant. The IVH was graded into four categories according to the modified Papile criteria (18). Mild IVH is defined as grade I or II, while severe IVH is defined as grade III or IV.

The secondary outcomes were the incidence of hypothermia, scleredema, hypoglycemia, perinatal respiratory diseases, apnea, pulmonary hemorrhage, severe asphyxia, Meconium aspiration syndrome of the newborn (MAS), persistent pulmonary hypertension of the newborn (PPHN), pneumothorax within seven days, respiratory failure, metabolic acidosis, circulatory failure, cardiopulmonary failure, necrotizing enterocolitis (NEC), hypoxic-ischemic encephalopathy (HIE), ROP and mortality. Hypothermia was defined as temperature < 36°C, measured by axillary method upon admission to NICU. Hypoglycemia was defined as a plasma glucose level of less than 40 mg/dl (2.2 mmol/L) upon admission to NICU. Perinatal respiratory diseases included pulmonary hemorrhage, severe asphyxia, PPHN, MAS, pneumothorax within seven days, respiratory failure, apnea.

Process measures that indicated the effects of DR management or contributed to the outcomes were tracked.

### Statistical analyses

IBM SPSS Statistics 27.0 and R 4.2.3 software were used for data analysis. Continuous data was expressed as medians (range) due to non-normal distribution. Mann–Whitney rank-sum tests was used to compare continuous and ranked data between groups. Counts were expressed as the numbers of cases and percentages. The Chi-square (chi^2^) test or the Fisher's exact test was used to compare the categorical data.

Outcomes variables were compared between two groups (non-DICU group and DICU group), binary logistic regressions were used to analyze univariate factors. Stratified analyses based on GA categories (<32 weeks, 32–33^+6^ weeks, 34–36^+6^ weeks) were performed to evaluate subgroup differences. Furthermore, we used propensity score matching (PSM) to align the distribution of observed baseline characters between two groups, thereby facilitating an efficient comparison of potential outcomes to assess sensitivity. Absolute Standardized Mean Difference (ASMD) was utilized to assess the balance of covariates before and after PSM. For adjusted analyses, we used the mixed-effects logistic regression (GLMER) model to analyze adjusted multivariate factors, the preferred model was selected based on ID. Multivariate analyses were conducted with “antenatal factors” or “perinatal factors” as random effects. Only factors significantly associated (*P* < 0.1) with each significant outcome were used for fitting multivariable models. Collinearities between variables to be included in the multivariable model were tested.

The stepwise regression was conducted by bidirectional elimination to identify the independent predictive factors. R 4.2.3 was used to plot the column plots of the predicted IVH model. Predictive performance was evaluated by the area under the receiver-operating characteristic (ROC) curve (AUC). We used calibration curves to represent the relationship between observed frequency and predicted probability. The Bootstrap method was used to repeat the sampling 1,000 times for internal validation. To assess the discriminative ability of the model, the Hosmer–Lemeshow test was conducted. The decision curve analysis (DCA) of the models was plotted to assess the clinical practicability. Differences were considered statistically significant at *P* < 0.05.

## Results

**Figure 2:**
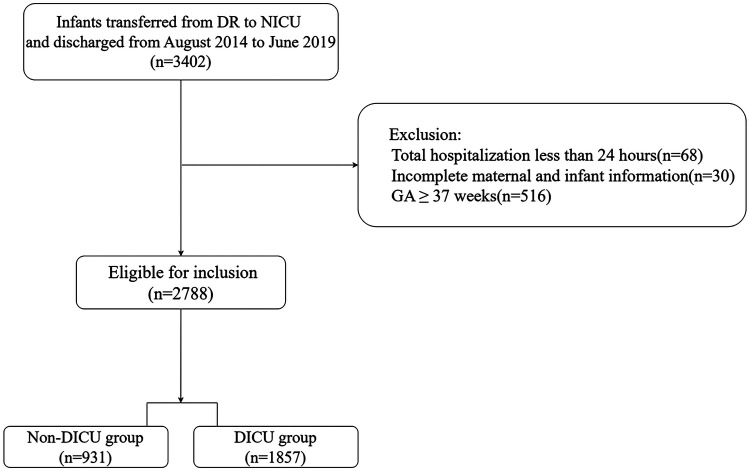
Flowchart of patient enrollment for this retrospective study.

During the period, 3,402 infants were transferred from DR to NICU. After applying our inclusion criteria, only 2,788 preterm infants (*n* = 931 for non-DICU and *n* = 1,857 for DICU group) were enrolled for this study (Figure 2).

### Demographic characteristics of the study

Baseline characteristics of the two groups (non-DICU and DICU group) are outlined in Table 1: For antenatal factors, there were no significant differences in the incidence of maternal age ≥ 35, antepartum hemorrhage, main maternal comorbidities ≥ 2, antibiotic use in pregnancy, placental abnormalities, grade III meconium stained amniotic fluid, umbilical cord abnormalities, fetal distress, cervical cerclage, obstetric anesthesia, caesarean and meconium aspiration syndrome between two groups (all *P* ≥ 0.05). However, the incidence of IVF-ET (*in vitro* fertilization embryo transfer), antenatal steroids, and abnormal labor stage was higher (*P* < 0.05) than that in the non-DICU group respectively, and the incidence of IAI (intraamniotic infection), GDM (gestational diabetes mellitus), HDPs (hypertensive disorders of pregnancy), PROM (premature rupture of membranes) and primiparity was lower (*P* < 0.05) than that in the non-DICU group, respectively. For perinatal factors, there were no significant differences in the incidence of male, multiple pregnancy and congenital abnormalities between the two groups (all *P* > 0.05), while there were significant differences in the proportion of GA and birth weight (BW) between two groups (all *P* < 0.05).

**Table 1:** Perinatal characteristics of preterm infants in the DICU group vs. the Non-DICU group.

Variables	Non-DICU (*N* = 931)	DICU (*N* = 1,857)	*P*
GA (week)			
<28	7 (0.7)	64 (3.4)	<0.001
28–33^+6^	442 (47.5)	908 (48.9)	
34–36^+6^	482 (51.8)	885 (47.6)	
BW (gram)			
<1,500	151 (16.2)	432 (23.3)	<0.001
1,500–2,499	676 (72.6)	1,232 (66.3)	
≥2,500	104 (11.2)	193 (10.4)	
Male	494 (53.1)	972 (52.4)	0.709
Multiple pregnancy	445 (47.8)	944 (50.8)	0.130
Congenital abnormalities	14 (1.5)	49 (2.6)	0.056
Maternal age ≥ 35	196 (21.1)	419 (22.6)	0.364
IVF-ET	227 (24.4)	567 (30.5)	<0.001
Antepartum hemorrhage	104 (11.2)	194 (10.4)	0.560
Antenatal steroids	773 (83.0)	1,623 (87.4)	0.002
IAI	25 (2.7)	12 (0.6)	<0.001
GDM	117 (12.6)	171 (9.2)	0.006
HDPs	58 (6.2)	65 (3.5)	<0.001
Main maternal comorbidities ≥ 2	255 (27.4)	499 (26.9)	0.786
Antibiotic use in pregnancy	399 (42.9)	781 (42.1)	0.687
Placental abnormalities	232 (24.9)	455 (24.5)	0.809
Grade III MSAF	29 (3.1)	41 (2.2)	0.149
Umbilical cord abnormalities	273 (29.3)	514 (27.7)	0.363
PROM	357 (38.3)	637 (34.3)	0.036
Fetal distress	105 (11.3)	192 (10.3)	0.449
Abnormal labor stage	39 (4.2)	150 (8.1)	<0.001
Cervical cerclage	4 (0.4)	22 (1.2)	0.050
Obstetric anesthesia	718 (77.1)	1,462 (78.7)	0.332
Primiparity	478 (51.3)	863 (46.5)	0.015
Caesarean	712 (76.5)	1,458 (78.5)	0.222
Meconium aspiration syndrome	3 (0.3)	2 (0.1)	0.341

Values were presented as M (IQR); M, median; IQR, interquartile range.

Abbreviations: DICU, delivery room intensive care unit; GA, gestational age; BW, birth weight; IVF-ET, *in vitro* fertilization embryo transfer; IAI, intraamniotic infection; GDM, gestational diabetes mellitus; HDPs, hypertensive disorders of pregnancy; MSAF, meconium stained amniotic fluid; PROM, premature rupture of membranes.

Process measures that indicated the effects of DR management or contributed to the outcomes were shown on Supplementary Tables S1–S3. These measures included variations in DR management, laboratory parameters on DICU admission and treatments in NICU.

### Outcomes

Unadjusted rates of outcomes between two groups were demonstrated in Table 2. Significant (*P* < 0.001) improvement was observed in primary outcomes, and there was higher incidence of infants without IVH in DICU group (87.4%) than in non-DICU group (58%). For the secondary outcomes, the DICU group had significant (*P* < 0.001) lower incidences of hypothermia (2%), neonatal scleredema (0.3%), apnea (19.4%), perinatal respiratory diseases (26.1%) and metabolic acidosis (4.7%) than non-DICU group (hypothermia: 6.0%, scleredema: 6.8%, apnea: 26.3%, perinatal respiratory diseases: 34.3%, metabolic acidosis: 17.6%). However, the DICU group had significant higher (*P* < 0.001) incidence of hypoglycemia than non-DICU group (12.9% vs. 5.5%). The remaining outcomes did not show any significant difference (*P* > 0.05).

**Table 2:** Unadjusted morbidities and mortality in the DICU group vs. the Non-DICU group.

Variables	Non-DICU (*N* = 931)	DICU (*N* = 1,857)	*P*
IVH			
None	540 (58.0)	1,623 (87.4)	<0.001
Mild IVH	342 (36.7)	194 (10.4)	
Severe IVH	49 (5.3)	40 (2.2)	
Hypothermia	56 (6.0)	38 (2.0)	<0.001
Scleredema	63 (6.8)	6 (0.3)	<0.001
Hypoglycemia	51 (5.5)	239 (12.9)	<0.001
Perinatal respiratory diseases	319 (34.3)	485 (26.1)	<0.001
Apnea	245 (26.3)	360 (19.4)	<0.001
Pulmonary hemorrhage	20 (2.1)	34 (1.8)	0.566
Severe asphyxia	8 (0.9)	10 (0.5)	0.319
MAS	4 (0.4)	3 (0.2)	0.351
PPHN	2 (0.2)	7 (0.4)	0.721
Pneumothorax within seven days	9 (1.0)	20 (1.1)	0.787
Respiratory failure	81 (8.7)	148 (8.0)	0.508
Metabolic acidosis	164 (17.6)	87 (4.7)	<0.001
Circulatory failure	34 (3.7)	47 (2.5)	0.096
Cardiopulmonary failure	97 (10.4)	164 (8.8)	0.175
NEC			
NONE	1,814 (97.7)	918 (98.6)	0.103
Grade I-II	28 (1.5)	8 (0.9)	
Grade III	15 (0.8)	5 (0.5)	
HIE	2 (0.2)	2 (0.1)	0.862
ROP	81 (8.7)	165 (8.9)	0.871
Mortality	13 (1.4)	14 (0.8)	0.102

Abbreviations: DICU, delivery room intensive care unit; IVH, intraventricular hemorrhage; MAS, meconium aspiration syndrome of the newborn; PPHN, persistent pulmonary hypertension of the newborn; NEC, necrotizing enterocolitis; HIE, hypoxic-ischemic encephalopathy; ROP, retinopathy of prematurity.

**Figure 3:**
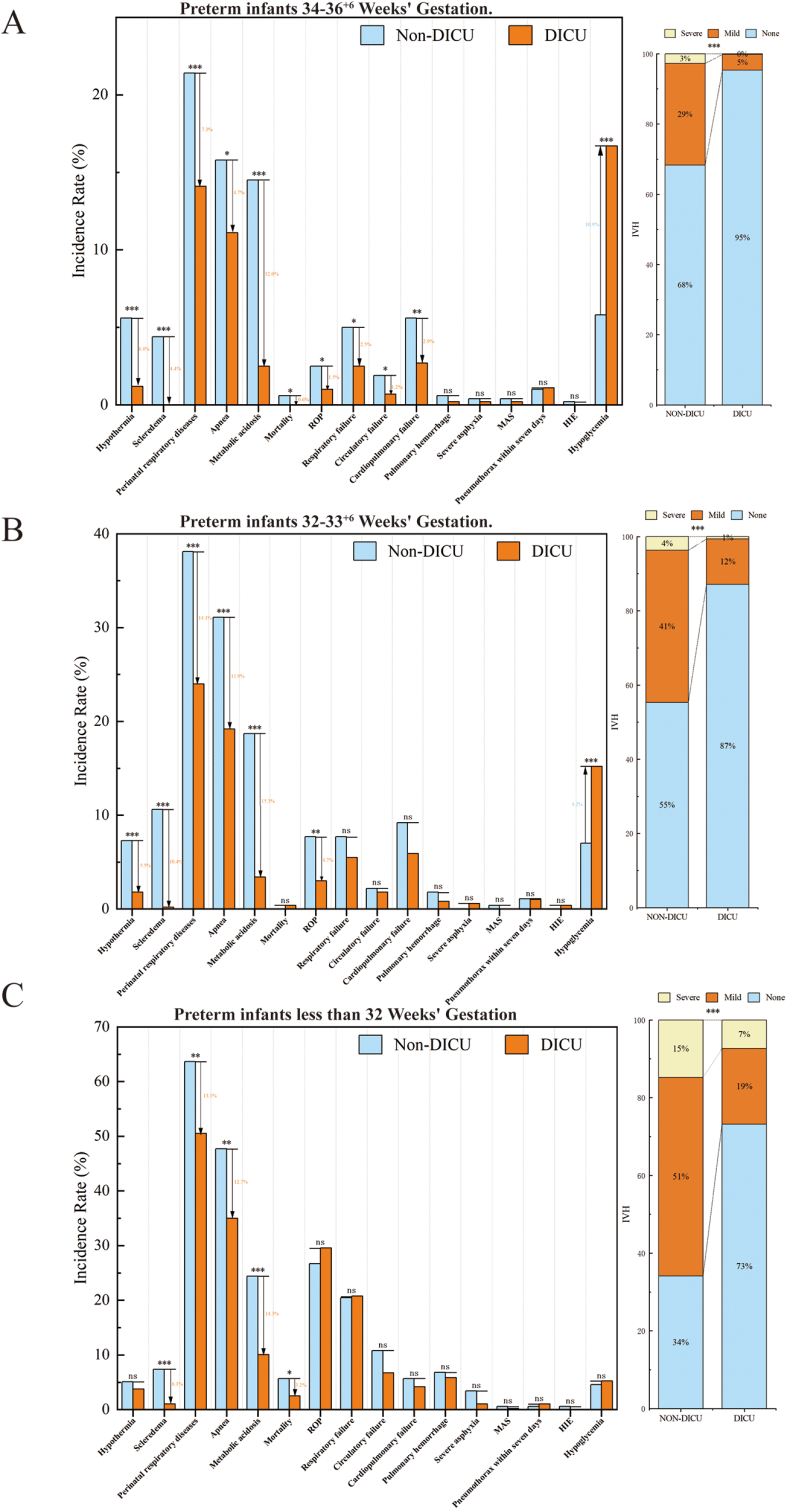
Morbidities and mortality in the DICU group vs. the Non-DICU group across different gestational age subgroups. **(A)** Infants less than 32 Weeks’ Gestation. **(B)** Infants 32–33^+6^ Weeks’ Gestation. **(C)** Infants 34–36^+6^ Weeks’ Gestation. Bar charts compare outcomes between the DICU and Non-DICU groups, with significant differences denoted by asterisks (*P < 0.05, **P < 0.01, ***P < 0.001).

Subgroup analyses across gestational age showed results consistent with overall findings, confirming the robustness of the interventions (Supplementary Tables S4–S6). For those less than 32 weeks of gestational age (Figure 3C), DICU group had significantly lower incidences of severe complications such as IVH (*P* < 0.001), scleredema (7.39% vs. 1.05%), perinatal respiratory diseases (63.64% vs. 50.52%), apnea (47.73% vs. 35.01%) and metabolic acidosis (24.43% vs. 10.06%), along with reduced mortality (5.68% vs. 2.52%, *P* = 0.047). Infants with smaller gestational ages exhibited higher baseline rates of complications and benefited most from interventions targeting severe conditions. For preterm infants aged 32–33^+6^ weeks (Figure 3B), apart from the aspects discussed above, improvements were observed in ROP (7.7% vs. 3.0%, *P* = 0.006). This subgroup demonstrated consistent benefits for moderate and severe complications. For preterm infants aged 34–36^+6^ weeks (Figure 3A), the DICU group showed significant reductions in hypothermia, scleredema, perinatal respiratory diseases, apnea, metabolic acidosis, mortality, ROP, respiratory failure, circulatory failure, and cardiopulmonary failure (all *P* < 0.05). This subgroup, while at lower baseline risk, benefited from broader improvements in more complications.

Under PSM with replacement, baseline characteristics were well balanced between the groups, as evidenced by absolute standardized differences (Supplementary Figure S1). The effect size indice for all covariates remained at 0.1. GA and BW distributions showed statistically significant differences between the two groups (*P* = 0.006 and *P* = 0.008, respectively). Furthermore, apart from IVF, abnormal labor stage and primiparity, other maternal and perinatal characteristics were balanced between the groups (*P* > 0.05) (Supplementary Table S7). In the matched analysis (Supplementary Table S8), results remained consistent with the unadjusted findings for most outcomes. The DICU group consistently demonstrated significantly lower incidences of IVH, hypothermia, scleredema, apnea, perinatal respiratory diseases, and metabolic acidosis, while a higher incidence of hypoglycemia was observed (all *P* < 0.001). Additionally, a new significant reduction in circulatory failure (*P* = 0.022) and mortality (*P* = 0.009) was identified.

Results from the GLMER model accounting for risk adjustment for antenatal and perinatal-level factors between two groups are shown in Table 3. We accounted for the following risk adjustment variables: GA, BW, IVF-ET, antenatal steroids, IAI, GDM, HDPs, PROM, abnormal labor stage and primiparity. The DICU group was associated with significantly lower odds of IVH [Odd ratio (OR): 0.16; 95%confidence interval (CI): 0.11–0.23], hypothermia (OR: 0.33; 95% CI: 0.21–0.50), apnea (OR: 0.60; 95%CI: 0.47–0.75), perinatal respiratory diseases (OR: 0.63; 95%CI: 0.52–0.75) and metabolic acidosis (OR: 0.24; 95% CI: 0.16–0.34) compared with non-DICU group (*P* < 0.001). There were no significant differences for scleredema (*P* = 0.216) and hypoglycemia (*P* = 0.268) between two groups.

**Table 3:** Adjusted odds ratio of morbidities in the DICU group vs. the Non-DICU group.

Outcomes	OR (95% CI)	*P*
IVH	0.16 (0.11,0.23)	<0.001
Hypothermia	0.33 (0.21,0.50)	<0.001
Scleredema	0.04 (0.00,6.30)	0.216
Hypoglycemia	2.65 (0.47,14.8)	0.268
Perinatal respiratory diseases	0.63 (0.52,0.75)	<0.001
Apnea	0.60 (0.47,0.75)	<0.001
Metabolic acidosis	0.24 (0.16,0.34)	<0.001

Abbreviations: OR, odd ratio; CI, confidence interval; DICU, delivery room intensive care unit; IVH, intraventricular hemorrhage.

### Risk factors in preterm infants with IVH and nomogram construction

**Figure 4:**
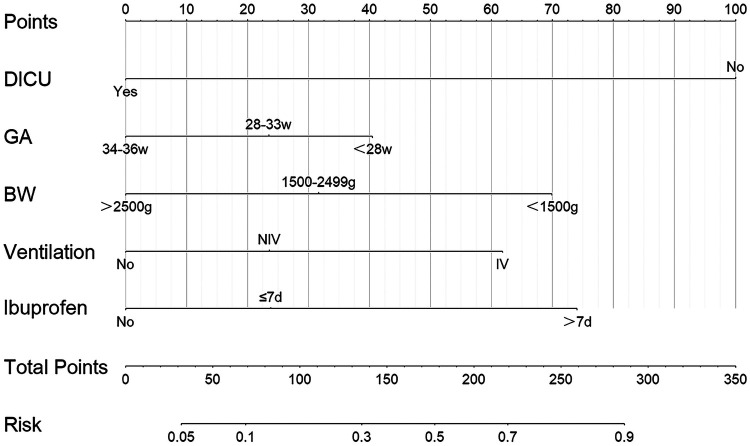
The nomogram for the early prediction model for IVH.

For the primary outcomes, 32 preterm infants were excluded due to incomplete data, after random sampling in a ratio of 7:3, 1929 and 827 infants were included in the training set and validation set, respectively. The characteristics data of patients in the two sets was given in Table 4. Indicators with significant differences between the two groups in the univariate analysis were selected, which included DICU, GA, BW, PS use, chest compression, ventilation mode within seven days, antibiotics use (d), ibuprofen use (d), hemoglobin (Hb) and PLT. They were included in the multivariate model, stepwise regression analysis revealed that DICU, GA, BW, ventilation mode within seven days and days for ibuprofen use were the independent predictors of IVH. Among them, DICU, GA and BW were independent protective factors, while ventilation mode within seven days and days for ibuprofen use were independent risk factors. The results of the logistic regression model were given in Table 5, all variables showed significant statistical differences. The IVH in DICU group risk nomogram was developed and was presented in Figure 4.

**Table 4:** Differences in characteristics for enrolled population between the training set and the validation set.

Variable	Total (*n* = 2,756)	train_set (*n* = 1,929)	valid_set (*n* = 827)	*P*
IVH	615 (22.31)	430 (22.29)	185 (22.37)	0.964
DICU	1,840 (66.76)	1,285 (66.61)	555 (67.11)	0.800
GA (week)				
<28	65 (2.36)	43 (2.23)	22 (2.66)	0.780
28–33^+6^	1,337 (48.51)	935 (48.47)	402 (48.61)	
34–36^+6^	1,354 (49.13)	951 (49.30)	403 (48.73)	
BW (gram)				
<1,500	573 (20.79)	397 (20.58)	176 (21.28)	0.806
1,500–2,499	1,887 (68.47)	1,328 (68.84)	559 (67.59)	
≥2,500	296 (10.74)	204 (10.58)	92 (11.12)	
PS use	146 (5.30)	94 (4.87)	52 (6.29)	0.129
Chest compression	147 (5.33)	111 (5.75)	36 (4.35)	0.134
Ventilation mode within 7 days				
None	1,231 (44.67)	861 (44.63)	370 (44.74)	0.992
Noninvasive ventilation	1,284 (46.59)	900 (46.66)	384 (46.43)	
Invasive ventilation	241 (8.74)	168 (8.71)	73 (8.83)	
Ibuprofen use				
None	2,689 (97.57)	1,889 (97.93)	800 (96.74)	0.091
≤7d	40 (1.45)	26 (1.35)	14 (1.69)	
>7d	27 (0.98)	14 (0.73)	13 (1.57)	
Antibiotics use				
None	912 (33.09)	637 (33.02)	275 (33.25)	0.978
≤7d	911 (33.06)	640 (33.18)	271 (32.77)	
>7d	933 (33.85)	652 (33.80)	281 (33.98)	
Hb (g/dl)	174.00 (30.00)	174.00 (30.00)	175.00 (30.00)	0.359
PLT (per ul)	242.00 (82.25)	243.00 (81.00)	242.00 (83.50)	0.792

Values were presented as M (IQR); M, median; IQR, interquartile range.

Abbreviations: IVH, intraventricular hemorrhage; DICU, delivery room intensive care unit; GA, gestational age; BW, birth weight; PS, pulmonary surfactant; CRP, C-reactive protein; Hb, hemoglobin; PLT, platelet.

**Table 5:** Predictors for the risk of IVH in the entire enrolled population.

Variables	cOR (95% CI)	*P*	aOR (95% CI)	*P*
DICU	0.20 (0.16–0.25)	<.001	0.14 (0.10–0.18)	<.001
GA (week)				
<28				
28–33^+6^	0.39 (0.21–0.72)	0.003	0.69 (0.33–1.44)	0.324
34–36^+6^	0.16 (0.09–0.30)	<.001	0.43 (0.19–0.94)	0.036
BW (gram)				
<1,500				
1,500–2,499	0.36 (0.28–0.46)	<.001	0.46 (0.34–0.64)	<.001
≥2,500	0.17 (0.10–0.27)	<.001	0.24 (0.13–0.44)	<.001
PS use	2.27 (1.48–3.49)	<.001		
Chest compression	3.63 (2.46–5.36)	<.001		
Ventilation within 7 days (highest level)				
None				
Noninvasive ventilation	1.57 (1.24–1.99)	<.001	1.60 (1.19–2.14)	0.002
Invasive ventilation	4.68 (3.29–6.66)	<.001	3.13 (1.94–5.06)	<.001
Antibiotics use (day)				
None	Reference			
≤7	1.12 (0.84–1.49)	0.442		
>7	2.27 (1.74–2.96)	<.001		
Ibuprofen use (day)				
None				
≤7	4.25 (1.95–9.26)	<.001	1.58 (0.60–4.16)	0.353
>7	6.55 (2.18–19.66)	<.001	4.41 (1.37–14.17)	0.013
Hb (g/dl)	0.99 (0.99–0.99)	0.005		
PLT (per ul)	0.99 (0.99–0.99)	0.002		

Abbreviations: cOR, crude odd ratio; CI, confidence interval; aOR, adjusted odd ratio; IVH, intraventricular hemorrhage; DICU, delivery room intensive care unit; GA, gestational age; BW, birth weight; PS, pulmonary surfactant; CRP, C-reactive protein; Hb, hemoglobin; PLT, platelet.

### Validation and efficacy of nomogram

**Figure 5:**
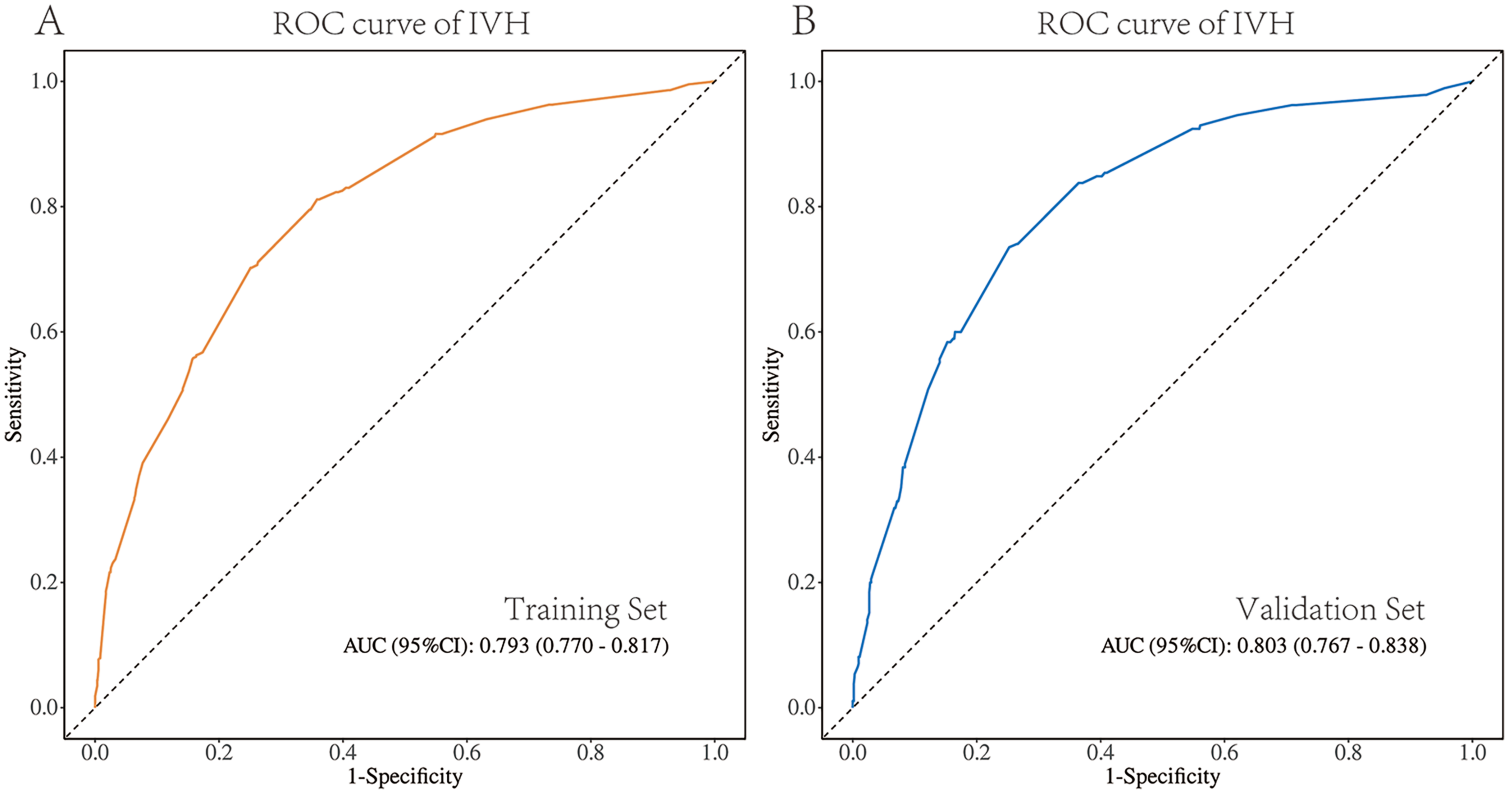
ROC validation for the IVH risk nomogram prediction. **(A)** ROC validation of the IVH risk nomogram prediction from the training set. **(B)** ROC validation of the IVH risk nomogram prediction from the validation set.

**Figure 6:**
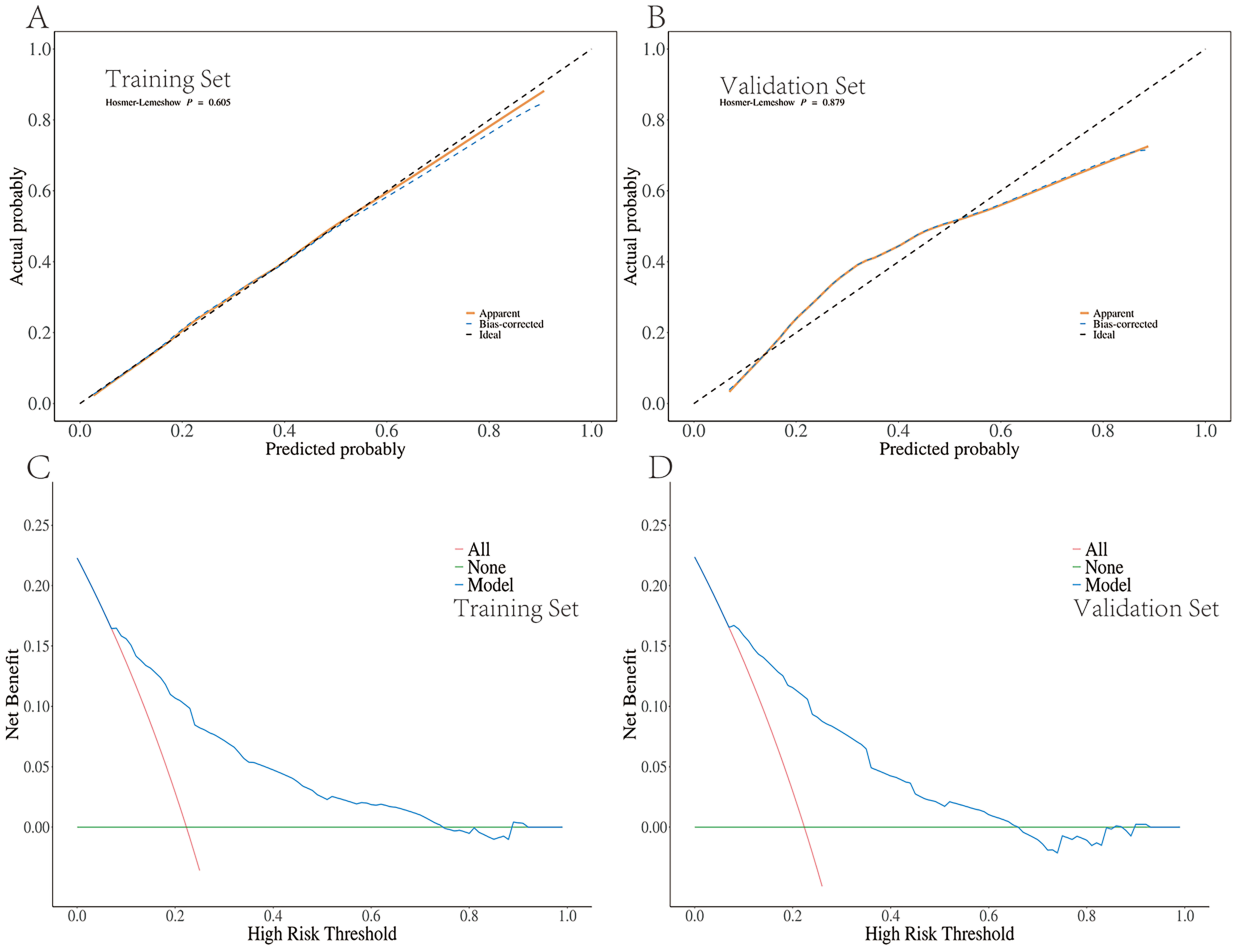
Calibration curves and decision curve analysis for the IVH risk nomogram prediction. **(A)** Calibration curves of the IVH risk nomogram prediction from the training set. **(B)** Calibration curves of the IVH risk nomogram prediction from the validation set. **(C)** Decision curve analysis for the IVH risk nomogram prediction from the training set. **(D)** Decision curve analysis for the IVH risk nomogram prediction from the validation set.

The pooled area under the ROC curve of the nomogram was 0.793 (95% CI: 0.770–0.817) in the training set and 0.803 (95% CI: 0.767–0.838) in the validation set (Figures 5A,B), which indicated moderately good predictive performance. The calibration curve of the nomogram to predict the IVH risk showed good consistency between the predicted and actual probabilities (Figures 6A,B). Hosmer-Lemeshow goodness of fit test indicated that the predictive models were well calibrated (Training set: *P* = 0.605; validation set: *P* = 0.879). The decision curve showed that it would be more accurate to use this nomogram in the current study to predict the risk of IVH when the risk threshold probability was between 2.5% and 87.1%, and between 3.3% and 82.3% in the validation set (Figures 6C,D). Within this range, the net benefit was comparable with several overlaps.

## Discussion

The key to successful treatment lies in the transformation of medical and nursing staff's treatment philosophy, shifting from the previous emphasis on “NICU-based treatment” to a focus on the “care, support, and treatment” model with an emphasis on early intervention. To our knowledge, this is the first study in China to demonstrate in a large population, that the forward shift in standardized team-based neonatal care practices has been conducted to optimize treatment resources and reduce the incidence of morbidity among high-risk preterm infants. The GA and BW of the transferred infants exhibited a declining trend, with an increasing proportion of infants born at <28 weeks and weighing <1,500 g, while since the exposure of DICU, the mortality rate among these infants decreased from 1.4% to 0.8%. By broadening the applicable GA range, we observed even more significant improvements in preterm infants aged >32 weeks of GA. Our transport team provided an accessible approach to better support fetal-to-neonatal transition, and significantly improve the outcomes of IVH, hypothermia, neonatal scleredema, apnea, perinatal respiratory diseases and metabolic acidosis. Furthermore, we have successfully developed a nomogram model that could effectively predict IVH, demonstrating robust internal validation consistency.

Our study expanded the population to include all infants with a GA of less than 34 weeks and critically ill infants less than 37 weeks. Traditionally, research has disproportionately focused on the prognosis of extremely preterm infants, often overlooking moderate and late preterm infants. These groups, despite having a lower incidence of adverse outcomes compared to extreme preterm infants, constitute approximately 80% of all preterm births and face significant risks that warrant attention due to their larger representation within the preterm population (19). In our study, we observed that DICU significantly reduced the incidence of key morbidities even among late preterm infants. Notably, DICU exposure had a profound impact on improving respiratory and circulatory support, with a marked reduction in the incidence of cardiovascular failure in this group. For these more mature preterm infants, earlier application of DICU concepts significantly improved both prognosis and quality of life. These findings challenge the traditional reliance on GA as the sole criterion for admission to the DICU. Late preterm infants with perinatal high-risk factors also benefit from multidisciplinary and collaborative management within the DICU. This suggests the necessity for early assessment and individualized treatment plans that consider the infant's overall health status rather than GA alone. The expansion of the research population had not only enhanced our understanding of early resuscitation management for preterm infants, but also provided crucial reference for clinicians. We underscore the critical importance of expanding research focus and clinical attention to moderately and late preterm infants, who have historically been underrepresented in studies despite their significant contribution to preterm morbidity and mortality.

With the intervention of the transport team, the incidence of hypothermia was significantly reduced from 6.0% to 2.8%, attributed to improved team cooperation and emphasize on maintaining warmth. A comparable improvement was observed in the reduced incidence of Scleredema, which decreased from 6.8% to 0.3% following the exposure of DICU. After adjusting at the patient's level between groups, DICU remains an independent protective factor against hypothermia. While, this study revealed that the incidence of hypoglycemia in preterm infants increased from 5.5% to 12.9% when compared DICU with non-DICU group. Based on age stratification results, the most significant rise in the incidence of hypoglycemia was observed among preterm infants aged between 34 and 36^+6^ weeks, escalating from 5.8% to 16.7%. After adjusting for the patients baseline character, it was found that the DICU exposure had no impact on blood glucose changes. We supposed that for preterm infants aged between 34 and 36^+6^ weeks, who did not require advanced life support casually, delayed establishment of venous access may result in hyperglycemia. This implied that for the future DICU team, neonatal nurse practitioners should promptly establish peripheral venous access during resuscitation in DRs to maintain stable blood glucose levels.

For the respiratory system, DICU group significantly reduced the incidence of apnea and perinatal respiratory diseases compared to the non-DICU group. Overall, after adjusting for patient factors through calibrated analyses, it was determined that the NICU exposure independently protective risks for metabolic acidosis, apnea, and even perinatal respiratory diseases. The prognosis of preterm infants with perinatal respiratory diseases is closely associated with early application of PS, appropriate oxygen use, and respiratory support (20). By conducting a comprehensive evaluation of the initial condition and implementing standardized respiratory management, it is possible to increase the number of preterm infants who are eligible for PS use in the DR and initiate adjunctive therapy at an earlier stage. Therefore, our study observed an increased use of PS and intubation in the DR within the DICU group, this is consistent with previous research (8, 21, 22). Furthermore, upon admission to the NICU, the DICU group facilitated early and continuous utilization of respiratory support through seamless transitions. Specifically, respiratory support (ventilation mode within seven days) was effectively implemented, predominantly in the proportion of non-invasive ventilation (NIV). The DICU group with involvement of respiratory therapists had a more standardized management of neonatal airway and T-tube. As the only entity in China with the qualification to train respiratory therapists, it holds significant advantages in enhancing patient care quality and reducing medical risks.

For the circulation system, we observed significant improvements in circulatory failure and cardiopulmonary failure for preterm infants aged between 34 and 36^+6^ Weeks' Gestation. Relevant studies have previously revealed that advanced resuscitation, including chest compressions, 100% oxygen supply in the DR, and potential use of epinephrine (23), were required for up to 15% of preterm infants (24). There was a notable reduction in the proportion of chest compressions through timely assessment and standardized resuscitation management. Upon entering the NICU, indicators showed a decrease in Hb levels, suggesting improved umbilical cord management by the NICU group and progress made towards reducing umbilical cord compression to mitigate later anemia risks.

For the nervous system, we selected IVH for analysis. Our findings confirm that IVH incidence is inversely proportional to gestational age (25), with neonates born at <32 weeks exhibiting the highest rates. However, across all gestational age groups, the DICU group consistently demonstrated lower IVH incidence compared to the non-DICU group, underscoring its protective effect regardless of maturity level. Notably, in the 34–36^+6^ weeks group, where the overall IVH risk is relatively low due to improved vascular stability, DICU further reduced IVH rates, highlighting its universal applicability. Moreover, the implementation of DICU was associated with significant reductions in both mild and severe IVH. Mild IVH incidence showed a marked decrease, suggesting that DICU not only prevents the onset of IVH but also mitigates its progression to severe forms.

We developed a nomogram prediction model for individualized prediction with good discrimination and calibration ability. We identified DICU exposure, GA, BW, ventilation mode within seven days, and days for ibuprofen use as independent predictors.

Positive pressure ventilation within seven days may contribute to an increased incidence of IVH due to hemodynamic changes induced by pressure fluctuations (26). However, the use of positive pressure ventilation is indispensable in the treatment of preterm infants. We conducted a comparative analysis of different ventilation modes and observed that the IVH incidence exhibited an upward trend with increasing levels of ventilatory pressure when compared to no ventilation. Furthermore, our findings highlighted invasive ventilation (IV) as having the most significant impact on IVH occurrence. This observation may be attributed to the inherent severity of the disease itself.

Our model results indicated an increased risk of IVH in preterm infants with prolonged use of ibuprofen; nevertheless, larger trials are necessary to assess its safety and efficacy. This may be attributed to the potential of long-term ibuprofen use in children with PDA to induce pulmonary hemorrhage, leading to inadequate systemic circulation and subsequent IVH. A meta-analysis comprising 9 studies demonstrated that ibuprofen treatment effectively reduced the incidence of PDA; however, no significant difference was observed in the occurrence of IVH compared to the untreated group, indicating a lack of evident neuroprotective effect (27).

In our study, the nomogram model once again confirmed the critical roles of gestational age and birth weight in the development of IVH. However, for neonatology teams facing preterm infants with fixed gestational ages and weights post-birth, the implementation of DICU serves as an independent protective factor against IVH. This underscores the significance of systematic respiratory and circulatory management during early resuscitation under the DICU framework. Through this model, neonatologists are guided to assess both the mother and infant comprehensively, identify key treatment priorities, and allocate team resources effectively. The inclusion of respiratory therapists ensures specialized evaluation and management of respiratory status, with a focus on optimizing FiO2 and PEEP settings to provide superior respiratory and airway support. Nurses play a crucial role not only in maintaining thermal stability and early glucose control but also in establishing venous access promptly to secure circulation, particularly for moderate and late preterm infants who are at heightened risk of glucose dysregulation. The standardized DICU management workflow we propose spans from pre-delivery preparation to seamless handovers upon NICU admission, fostering improved collaboration among multidisciplinary teams, including obstetricians and families, while enhancing outcomes for IVH and other complications.

The nomogram serves as a practical tool, offering real-time risk predictions and actionable guidance. In facilities with limited staff or expertise, it supports less-experienced providers with evidence-based recommendations, enhancing consistency in decision-making and overall care quality. By facilitating early interventions, such as optimizing ventilation strategies and stabilizing glucose levels, it demonstrates significant potential to prevent IVH and improve neonatal outcomes across a wide range of clinical settings.

### Strength

Faced with a significant number of premature births, there is immense pressure on specialized pediatric transport teams for training and resources required. A thorough understanding of infant characteristics and identification of potential risk factors are crucial to alter specific treatment decisions. As the first large-scale study of its kind in China, our center has established an early resuscitation model starting from the delivery room (DR), providing a framework that can be extended to other regions in China and even internationally. Notably, this model achieves these improvements without adding significant cost burdens, making it particularly beneficial for regions facing similar challenges. The protocol highlighted the importance of early intervention based on standardized team resuscitation resources.

A key strength of our approach lies in the integration of respiratory therapists, which has significantly enhanced respiratory management during early resuscitation. Furthermore, we emphasize the broader application of resuscitation strategies across a wider spectrum of GA, supported by favorable outcomes, challenging the traditional reliance on GA as the primary determinant of prognosis for moderate and late preterm infants.

For the primary outcome, our study identified critical predictive factors associated with the implementation of DICU, providing valuable insights for early prevention and clinical intervention strategies. These findings highlight the potential of these strategies to effectively reduce IVH incidence, with high accuracy and consistency confirmed through robust internal validation.

### Limitations

This study has several limitations. First, as a single-center study, the findings may not be generalizable to other clinical settings. It would be more advantageous to incorporate relevant advancements in alternative centers to enhance representation and increase the external validity of the results. Second, while this study focuses on short-term outcomes, it lacks data on the long-term impact of DICU, including neurodevelopmental and respiratory outcomes, which are critical in evaluating the sustained benefits of early interventions. Future research should incorporate longitudinal designs to explore how DICU influences long-term health trajectories, particularly with regard to conditions such as bronchopulmonary dysplasia and necrotizing enterocolitis. Furthermore, the time span of 2014 to 2019 was selected due to the completeness of available data and the implementation of the team intervention in 2016. Future research will aim to extend this analysis by incorporating more data from 2020 onwards, which will allow for a more comprehensive assessment of the long-term impact of the intervention. Thirdly, this study does not include a cost-effectiveness analysis of the DICU model compared to standard NICU care. Such an evaluation is essential for assessing its feasibility and promoting its adoption, especially in resource-constrained settings. Finally, the model's predictive accuracy could be further enhanced by integrating more continuous and routine hospital-based indicators, ensuring broader applicability in both advanced and resource-constrained settings.

## Conclusions

The transportation of critically ill pediatric patients requires a trained professional team and substantial medical resources. This study emphasized the shift in early intervention concepts, highlighting the need for more accurate identification of preterm infant characteristics and timely provision of respiratory and circulatory support as primary “assistance” measures to continuously improve the survival rate and quality of life for both early and mid-term preterm infants. The standardized DICU approach had significantly reduced the incidence of hypothermia, apnea, perinatal respiratory diseases, metabolic acidosis and IVH. Furthermore, notable progress has been achieved in utilizing positive pressure support, PS and administering antibiotics. DICU exposure, GA, BW, ventilation mode within seven days, and days for ibuprofen use were independent predictors of IVH. The prediction model created based on these indicators had high accuracy, sensitivity, consistency, and practicability in predicting IVH, which will be helpful for clinicians to promptly determine, appropriately diagnose, and treat the disease to improve its prognosis. Furthermore, our study contributes to the existing body of research on perinatal care by providing evidence suggesting the potential benefits of extending gestational age (GA) to 34 weeks or below on the prognosis of preterm infants. We also highlight that GA alone should not be considered the sole criterion when assessing neonatal outcomes.

## Data Availability

The original contributions presented in the study are included in the article/Supplementary Material, further inquiries can be directed to the corresponding author.
