# Supplementary material for: Reforming early intervention for premature infants: insights into integrated nursing and medical care in Western China
Source: Front Pediatr. 2024 Dec 24;12:1469757. doi: 10.3389/fped.2024.1469757 (PMC11703874; doi:10.3389/fped.2024.1469757)
Supplement: Supplementary file 1 [file Datasheet1.docx]

**Supplementary Figure 1.** **Absolute standardized differences for baseline covariates comparing DICU to Non-DICU subjects in the original and the matched sample.
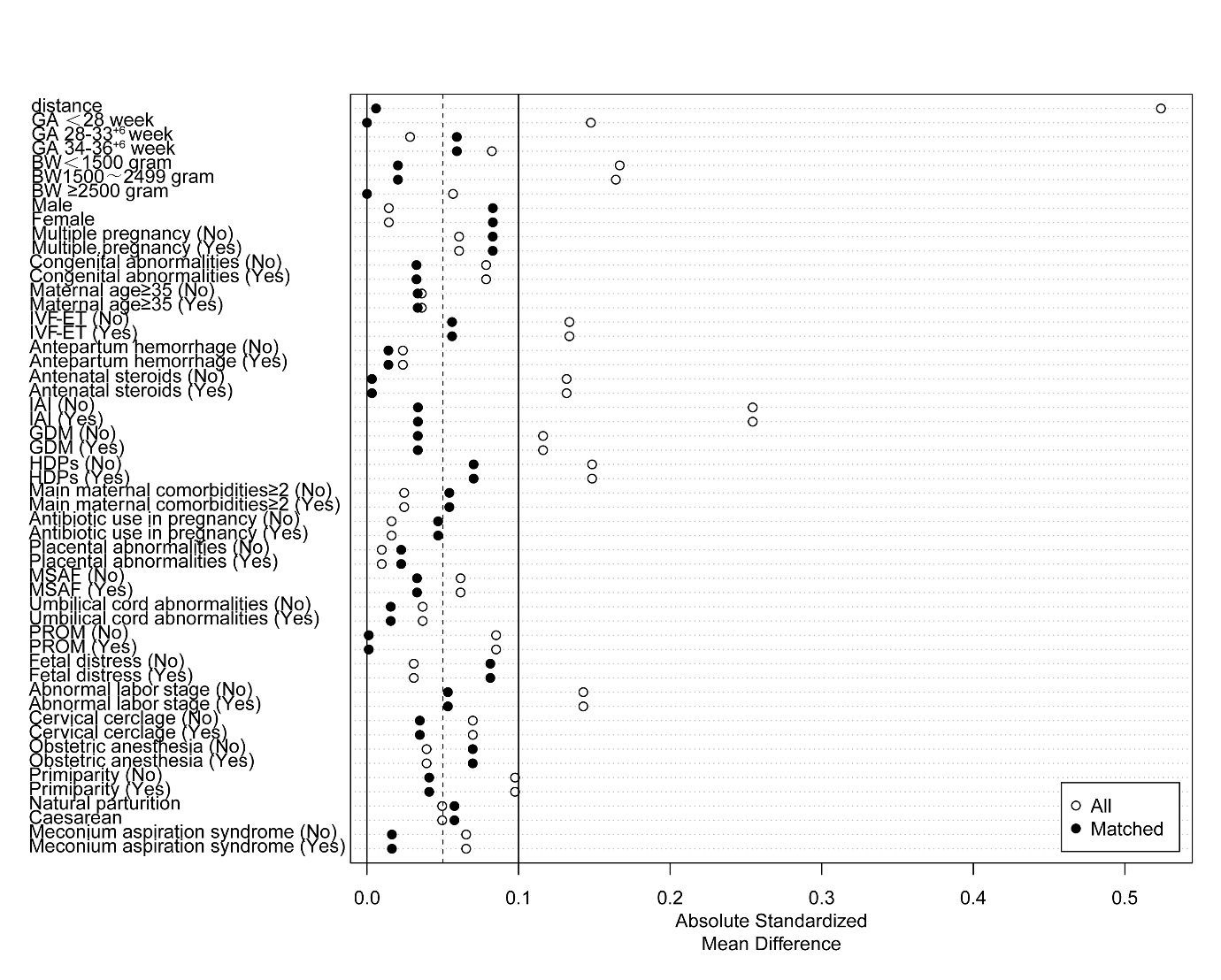
**

**Supplementary Tables**

| **TableS1.Delivery room management** | | | |
| --- | --- | --- | --- |
|  | Non-DICU | DICU | *P* |
|  | (N=931) | (N=1857) |  |
| Oxygen Use | 229(24.6) | 1024(55.1) | ＜0.001 |
| FiO2 |  |  |  |
| None | 696(100.0) | 832(65.0) | ＜0.001 |
| 21%-30% | 0(0.0) | 319(24.9) |  |
| 31%-40% | 0(0.0) | 63(4.9) |  |
| ＞40% | 0(0.0) | 66(5.2) |  |
| Tracheal intubation | 3(0.3) | 111(6.3) | ＜0.001 |
| Tracheal intubation (min) |  |  |  |
| None | 867(99.7) | 1654(93.7) | ＜0.001 |
| ＜1 | 2(0.2) | 91(5.2) |  |
| 1-5 | 1(0.1) | 17(0.9) |  |
| 5-10 | 0(0.0) | 2(0.1) |  |
| ＞10 | 0(0.0) | 1(0.1) |  |
| PS use | 5(0.5) | 146(7.9) | ＜0.001 |
| Chest compression | 88(9.5) | 62(3.3) | ＜0.001 |
| Epinephrine use | 9(1.0) | 12(0.6) | 0.356 |
| *Abbreviations: |  |  |  |
| DICU: delivery room intensive care unit;  FiO2: Fraction of inspiration O_2_; PS: Pulmonary surfactant | | | |

| **TableS2.Laboratory parameters on NICU admission** | | | |
| --- | --- | --- | --- |
|  | Non-DICU | DICU | *P* |
|  | (N=931) | (N=1857) |  |
| PH |  |  |  |
| ＜7.00 | 6(1.2) | 3(0.2) | ＜0.001 |
| 7.00-7.19 | 41(7.9) | 62(4.4) |  |
| ≥7.20 | 471(90.9) | 1334(95.4) |  |
| BE |  |  |  |
| ＜-16 | 4(0.8) | 10(0.7) | 1.000 |
| ≥-16 | 512(99.2) | 1389(99.3) |  |
| Lac |  |  |  |
| ＞5 | 36(7.1) | 73(5.2) | ＜0.001 |
| 2-5 | 253(49.5) | 357(25.6) |  |
| ＜2 | 222(43.4) | 965(69.2) |  |
| WBC count (6-8h) | 10.1(5.6) | 9.9(5.45) | 0.065 |
| Hb (g/dl) | 50.2(7.9) | 174(31.5) | ＜0.001 |
| HCT (%) | 50.20(7.90) | 50.65(8.4) | 0.082 |
| PLT (per ul) | 232(78.5) | 249(83) | ＜0.001 |
| CRP≥8 (mg/dl) | 58(3.2) | 23(2.5) | 0.348 |
| Blood culture (+) | 4(0.6) | 8(0.4) | 0.840 |
| Values were presented as M(IQR);  M: Median; IQR: Interquartile range | | | |
| *Abbreviations: | | | |
| NICU: neonatal intensive care unit  DICU: delivery room intensive care unit | | | |
| WBC: White blood cell; Hb: Hemoglobin  HCT: Hematocrit; PLT: Platelet; CRP: C-reactive protein | | | |

| **TableS3.Treatment in NICU** | | | |
| --- | --- | --- | --- |
|  | Non-DICU | DICU | *P* |
|  | (N=931) | (N=1857) |  |
| PS use(h) |  |  |  |
| None | 757(81.7) | 1467(79.1) | 0.106 |
| ≤6 | 139(15.0) | 304(16.4) |  |
| ＞6 | 30(3.3) | 84(4.5) |  |
| Ventilation mode within 7 days |  |  |  |
| None | 515(55.3) | 723(38.9) | ＜0.001 |
| Noninvasive ventilation | 340(36.5) | 961(51.8) |  |
| Invasive ventilation | 76(8.2) | 173(9.3) |  |
| Mild hypothermia therapy | 1(0.1) | 0(0.0) | 0.334 |
| Antibiotics use |  |  |  |
| None | 333(35.8) | 587(31.7) | ＜0.001 |
| Single | 575(61.7) | 854(46.1) |  |
| Double | 23(2.5) | 411(22.2) |  |
| Antibiotics use (d) |  |  |  |
| None | 333(35.8) | 587(31.7) | ＜0.001 |
| ≤7 | 253(27.2) | 673(36.3) |  |
| ＞7 | 345(37.0) | 592(32.0) |  |
| Ibuprofen use (d) |  |  |  |
| None | 914(98.2) | 1801(97.0) | 0.016 |
| ≤7 | 15(1.6) | 31(1.7) |  |
| ＞7 | 2(0.2) | 25(1.3) |  |
| Saline volume expansion |  |  |  |
| None | 909(97.7) | 1827(98.4) | 0.387 |
| Once | 17(1.8) | 23(1.2) |  |
| More than twice | 5(0.5) | 7(0.4) |  |
| Vasoactive substances use (d) |  |  |  |
| None | 909(97.6) | 1820(98.0) | 0.792 |
| ＜3 | 15(1.6) | 26(1.4) |  |
| 3-7 | 7(0.8) | 10(0.5) |  |
| ＞7 | 0(0.0) | 1(0.1) |  |
| *Abbreviations:  DICU: delivery room intensive care unit; PS: Pulmonary surfactant | | | |

**TableS4. Morbidities and mortality in the DICU group vs. the Non-DICU group for preterm infants less than 32 Weeks' Gestation.**

| Variables | Non-DICU  (n = 176) | DICU  (n = 477) | *P* |
| --- | --- | --- | --- |
|  |  |  |  |
| IVH |  |  |  |
| None | 60 (34.09) | 349 (73.17) | <.001 |
| Mild IVH | 90 (51.14) | 93 (19.50) |  |
| Severe IVH | 26 (14.77) | 35 (7.34) |  |
| Hypothermia | 9 (5.11) | 18 (3.77) | 0.445 |
| Scleredema | 13 (7.39) | 5 (1.05) | <.001 |
| Hypoglycemia | 8 (4.55) | 25 (5.24) | 0.719 |
| Perinatal respiratory diseases | 112 (63.64) | 241 (50.52) | 0.003 |
| Apnea | 84 (47.73) | 167 (35.01) | 0.003 |
| Pulmonary hemorrhage | 12 (6.82) | 28 (5.87) | 0.286 |
| Severe asphyxia | 6 (3.41) | 5 (1.05) | 0.082 |
| MAS | 1 (0.57) | 1 (0.21) | 0.467 |
| PPHN | 2 (1.14) | 7 (1.47) | 1.000 |
| Pneumothorax within seven days | 1 (0.57) | 5 (1.05) | 0.914 |
| Respiratory failure | 36 (20.45) | 99 (20.75) | 0.933 |
| Metabolic acidosis | 43 (24.43) | 48 (10.06) | <.001 |
| Circulatory failure | 19 (10.80) | 32 (6.71) | 0.084 |
| Cardiopulmonary failure | 10 (5.68) | 20 (4.19) | 0.420 |
| NEC |  |  |  |
| NONE | 170 (96.59) | 451 (94.55) | 0.634 |
| Grade I-II | 3 (1.70) | 15 (3.14) |  |
| Grade III | 3 (1.70) | 11 (2.31) |  |
| HIE | 1 (0.57) | 0 (0.00) | 0.270 |
| ROP | 47 (26.70) | 141 (29.56) | 0.610 |
| Mortality | 10 (5.68) | 12 (2.52) | 0.047 |
| *Abbreviations: | | | |
| DICU: delivery room intensive care unit; IVH: Intraventricular hemorrhage | | | |
| MAS: Meconium aspiration syndrome of the newborn | | | |
| PPHN: persistent pulmonary hypertension of the newborn; NEC: Necrotizing enterocolitis | | | |
| HIE: Hypoxic-ischemic encephalopathy; ROP: Retinopathy of prematurity | | | |

**TableS5. Morbidities and mortality in the DICU group vs. the Non-DICU group for preterm infants 32-33^+6^ Weeks' Gestation.**

|  | Non-DICU | DICU | *P* |
| --- | --- | --- | --- |
|  | (N=273) | (N=495) |  |
| IVH |  |  |  |
| None | 151(55.3) | 431(87.1) | ＜0.001 |
| Mild IVH | 112(41.0) | 61(12.3) |  |
| Severe IVH | 10(3.7) | 3(0.6) |  |
| Hypothermia | 20(7.3) | 9(1.8) | ＜0.001 |
| Scleredema | 29(10.6) | 1(0.2) | ＜0.001 |
| Hypoglycemia | 19(7.0) | 75(15.2) | ＜0.001 |
| Perinatal respiratory diseases | 104(38.1) | 119(24.0) | ＜0.001 |
| Apnea | 85(31.1) | 95(19.2) | ＜0.001 |
| Pulmonary hemorrhage | 5(1.8) | 4(0.8) | 0.362 |
| Severe asphyxia | 0(0.0) | 3(0.6) | 0.556 |
| MAS | 1(0.4) | 0(0.0) | 0.355 |
| PPHN | 0(0.0) | 0(0.0) |  |
| Pneumothorax within seven days | 3(1.1) | 5(1.0) | 1.000 |
| Respiratory failure | 21(7.7) | 27(5.5) | 0.284 |
| Metabolic acidosis | 51(18.7) | 17(3.4) | ＜0.001 |
| Circulatory failure | 6(2.2) | 9(1.8) | 0.927 |
| Cardiopulmonary failure | 25(9.2) | 29(5.9) | 0.118 |
| NEC |  |  |  |
| NONE | 269(98.5) | 486(98.2) | 0.715 |
| Grade I-II | 4(1.5) | 6(1.2) |  |
| Grade III | 0(0.0) | 3(0.6) |  |
| HIE | 0(0.0) | 2(0.4) | 0.541 |
| ROP | 21(7.7) | 15(3.0) | 0.006 |
| Mortality | 0(0.0) | 2(0.4) | 0.541 |
| *Abbreviations: | | | |
| DICU: delivery room intensive care unit; IVH: Intraventricular hemorrhage | | | |
| MAS: Meconium aspiration syndrome of the newborn | | | |
| PPHN: persistent pulmonary hypertension of the newborn; NEC: Necrotizing enterocolitis | | | |
| HIE: Hypoxic-ischemic encephalopathy; ROP: Retinopathy of prematurity | | | |

**TableS6. Morbidities and mortality in the DICU group vs. the Non-DICU group for preterm infants 34-36^+6^ Weeks' Gestation.**

|  | Non-DICU | | DICU | *P* |
| --- | --- | --- | --- | --- |
|  | (N=482) | | (N=885) |  |
| IVH |  | |  |  |
| None | 329(68.3) | | 843(95.3) | ＜0.001 |
| Mild IVH | 140(29.0) | | 40(4.5) |  |
| Severe IVH | 13(2.7) | | 2(0.2) |  |
| Hypothermia | 27(5.6) | 11(1.2) | | ＜0.001 |
| Scleredema | 21(4.4) | 0(0.0) | | ＜0.001 |
| Hypoglycemia | 28(5.8) | 148(16.7) | | ＜0.001 |
| Perinatal respiratory diseases | 103(21.4) | 125(14.1) | | ＜0.001 |
| Apnea | 76(15.8) | 98(11.1) | | 0.013 |
| Pulmonary hemorrhage | 3(0.6) | 2(0.2) | | 0.352 |
| Severe asphyxia | 2(0.4) | 2(0.2) | | 0.617 |
| MAS | 2(0.4) | 2(0.2) | | 0.617 |
| PPHN | 0(0.0) | | 0(0.0) |  |
| Pneumothorax within seven days | 5(1.0) | | 10(1.1) | 0.875 |
| Respiratory failure | 24(5.0) | | 22(2.5) | 0.015 |
| Metabolic acidosis | 70(14.5) | | 22(2.5) | ＜0.001 |
| Circulatory failure | 9(1.9) | | 6(0.7) | 0.044 |
| Cardiopulmonary failure | 27(5.6) | | 24(2.7) | 0.007 |
| NEC |  | |  |  |
| NONE | 479(99.4) | | 877(99.1) | 0.582 |
| Grade I-II | 1(0.2) | | 7(0.8) |  |
| Grade III | 2(0.4) | | 1(0.1) |  |
| HIE | 1(0.2) | | 0(0.0) | 0.353 |
| ROP | 12(2.5) | | 9(1.0) | 0.034 |
| Mortality | 3(0.6) | | 0(0.0) | 0.044 |
| *Abbreviations: | | | | |
| DICU: delivery room intensive care unit; IVH: Intraventricular hemorrhage | | | | |
| MAS: Meconium aspiration syndrome of the newborn | | | | |
| PPHN: persistent pulmonary hypertension of the newborn; NEC: Necrotizing enterocolitis | | | | |
| HIE: Hypoxic-ischemic encephalopathy; ROP: Retinopathy of prematurity | | | | |

**TableS7. Perinatal characteristics of preterm infants in the DICU group vs. the Non-DICU group after propensity score matching.**

| Variables | Non-DICU  (n = 597) | DICU  (n = 1857) | *P* |
| --- | --- | --- | --- |
|  |  |  |  |
| GA (week) |  |  | 0.006 |
| ＜28 | 6 (1.01) | 64 (3.45) |  |
| 28-33^+6^ | 290 (48.58) | 908 (48.90) |  |
| 34-36^+6^ | 301 (50.42) | 885 (47.66) |  |
| BW (gram) |  |  | 0.008 |
| ＜1500 | 108 (18.09) | 432 (23.26) |  |
| 1500～2499 | 489 (81.91) | 1425 (76.74) |  |
| Male | 298 (49.92) | 972 (52.34) | 0.302 |
| Multiple pregnancy | 290 (48.58) | 944 (50.83) | 0.337 |
| Congenital abnormalities | 12 (2.01) | 52 (2.80) | 0.292 |
| Maternal age≥35 | 126 (21.11) | 421 (22.67) | 0.424 |
| IVF-ET | 157 (26.30) | 567 (30.53) | 0.048 |
| Antepartum hemorrhage | 64 (10.72) | 194 (10.45) | 0.850 |
| Antenatal steroids | 503 (84.25) | 1623 (87.40) | 0.050 |
| IAI | 6 (1.01) | 12 (0.65) | 0.537 |
| GDM | 69 (11.56) | 171 (9.21) | 0.093 |
| HDPs | 27 (4.52) | 65 (3.50) | 0.253 |
| Main maternal comorbidities≥2 | 166 (27.81) | 509 (27.41) | 0.851 |
| Antibiotic use in pregnancy | 252 (42.21) | 781 (42.06) | 0.947 |
| Placental abnormalities | 162 (27.14) | 455 (24.50) | 0.197 |
| Grade III MSAF | 14 (2.35) | 41 (2.21) | 0.844 |
| Umbilical cord abnormalities | 172 (28.81) | 514 (27.68) | 0.592 |
| PROM | 227 (38.02) | 637 (34.30) | 0.098 |
| Fetal distress | 62 (10.39) | 192 (10.34) | 0.974 |
| Abnormal labor stage | 29 (4.86) | 150 (8.08) | 0.008 |
| Cervical cerclage | 4 (0.67) | 22 (1.18) | 0.285 |
| Obstetric anesthesia | 471 (78.89) | 1462 (78.73) | 0.932 |
| Primiparity | 305 (51.09) | 863 (46.47) | 0.049 |
| Caesarean | 467 (78.22) | 1458 (78.51) | 0.881 |
| Meconium aspiration syndrome | 1 (0.17) | 2 (0.11) | 0.567 |
| Values were presented as M(IQR); M: Median; IQR: Interquartile range | | | |
| *Abbreviations: | | | |
| DICU: delivery room intensive care unit; GA: gestational age; BW: birth weight | | | |
| IVF-ET: In vitro fertilization embryo transfer; IAI: intraamniotic infection | | | |
| GDM: Gestational diabetes mellitus; HDPs: hypertensive disorders of pregnancy | | | |
| MSAF: meconium stained amniotic fluid; PROM: Premature rupture of membranes | | | |

**TableS8. Unadjusted morbidities and mortality in the DICU group vs. the Non-DICU group after propensity score matching.**

| Variables | Non-DICU  (n = 597) | DICU  (n = 1857) | *P* | |
| --- | --- | --- | --- | --- |
|  |  |  |  |  |
| IVH |  |  |  | |
| None | 350 (58.63) | 1623 (87.40) | <.001 | |
| Mild IVH | 210 (35.18) | 194 (10.45) |  |  |
| Severe IVH | 37 (6.20) | 40 (2.15) |  |  |
| Hypothermia | 43 (7.20) | 38 (2.05) | <.001 | |
| Scleredema | 37 (6.20) | 6 (0.32) | <.001 | |
| Hypoglycemia | 35 (5.86) | 239 (12.87) | <.001 | |
| Perinatal respiratory diseases | 217 (36.35) | 485 (26.12) | <.001 | |
| Apnea | 169 (28.31) | 360 (19.39) | <.001 | |
| Pulmonary hemorrhage | 13 (2.18) | 34 (1.83) | 0.723 | |
| Severe asphyxia | 7 (1.17) | 10 (0.54) | 0.180 | |
| MAS | 2 (0.34) | 3 (0.16) | 0.767 | |
| PPHN | 2 (0.34) | 7 (0.38) | 1.000 | |
| Pneumothorax within seven days | 6 (1.01) | 20 (1.08) | 0.881 | |
| Respiratory failure | 52 (8.71) | 148 (7.97) | 0.565 | |
| Metabolic acidosis | 110 (18.43) | 87 (4.68) | <.001 | |
| Circulatory failure | 26(4.36) | 47(2.53) | 0.022 | |
| Cardiopulmonary failure | 14 (2.35) | 31 (1.67) | 0.284 | |
| NEC |  |  |  | |
| NONE | 589 (98.66) | 1814 (97.68) | 0.313 | |
| Grade I-II | 6 (1.01) | 28 (1.51) |  |  |
| Grade III | 2 (0.34) | 15 (0.81) |  |  |
| HIE | 1 (0.17) | 2 (0.11) | 0.567 | |
| ROP | 60 (10.05) | 165 (8.89) | 0.396 | |
| Mortality | 12 (2.01) | 14 (0.75) | 0.009 | |
| *Abbreviations: | | | |  |
| DICU: delivery room intensive care unit; IVH: Intraventricular hemorrhage | | | |  |
| MAS: Meconium aspiration syndrome of the newborn | | | |  |
| PPHN: persistent pulmonary hypertension of the newborn; NEC: Necrotizing enterocolitis | | | |  |
| HIE: Hypoxic-ischemic encephalopathy; ROP: Retinopathy of prematurity | | | |  |

**TableS9.STROBE Reporting guideline checklist**

|  | Item No | Recommendation | Location where item is reported |
| --- | --- | --- | --- |
| **Title and abstract** | 1 | (*a*) Indicate the study’s design with a commonly used term in the title or the abstract | Pg. 1 |
|  |  | (*b*) Provide in the abstract an informative and balanced summary of what was done and what was found | Pg. 2 |
| Introduction | | |  |
| Background/rationale | 2 | Explain the scientific background and rationale for the investigation being reported | Pgs. 4-6 |
| Objectives | 3 | State specific objectives, including any prespecified hypotheses | Pg. 6 |
| Methods | | |  |
| Study design | 4 | Present key elements of study design early in the paper | Pgs. 7-11 |
| Setting | 5 | Describe the setting, locations, and relevant dates, including periods of recruitment, exposure, follow-up, and data collection | Pgs. 7 |
| Participants | 6 | (*a*) Give the eligibility criteria, and the sources and methods of selection of participants. Describe methods of follow-up | Pg. 7 |
|  |  | (*b*) For matched studies, give matching criteria and number of exposed and unexposed | / |
| Variables | 7 | Clearly define all outcomes, exposures, predictors, potential confounders, and effect modifiers. Give diagnostic criteria, if applicable | Pgs. 8-11 |
| Data sources/ measurement | 8* | For each variable of interest, give sources of data and details of methods of assessment (measurement). Describe comparability of assessment methods if there is more than one group | Pgs. 10-11 |
| Bias | 9 | Describe any efforts to address potential sources of bias | Pg. 11-12 |
| Study size | 10 | Explain how the study size was arrived at | / |
| Quantitative variables | 11 | Explain how quantitative variables were handled in the analyses. If applicable, describe which groupings were chosen and why | Pgs. 11-12 |
| Statistical methods | 12 | (*a*) Describe all statistical methods, including those used to control for confounding | Pgs. 11-12 |
|  |  | (*b*) Describe any methods used to examine subgroups and interactions | Pgs. 11-12 |
|  |  | (*c*) Explain how missing data were addressed | / |
|  |  | (*d*) If applicable, explain how loss to follow-up was addressed | / |
|  |  | (*e*) Describe any sensitivity analyses | Pgs. 11-12 |
| Results | | |  |
| Participants | 13* | (a) Report numbers of individuals at each stage of study—eg numbers potentially eligible, examined for eligibility, confirmed eligible, included in the study, completing follow-up, and analysed | Pg. 13 |
|  |  | (b) Give reasons for non-participation at each stage | Pg. 13 |
|  |  | (c) Consider use of a flow diagram | Pg. 13 |
| Descriptive data | 14* | (a) Give characteristics of study participants (eg demographic, clinical, social) and information on exposures and potential confounders | Pg. 13 |
|  |  | (b) Indicate number of participants with missing data for each variable of interest | / |
|  |  | (c) Summarise follow-up time (eg, average and total amount) | / |
| Outcome data | 15* | Report numbers of outcome events or summary measures over time | Pgs. 14-15 |
| Main results | 16 | (*a*) Give unadjusted estimates and, if applicable, confounder-adjusted estimates and their precision (eg, 95% confidence interval). Make clear which confounders were adjusted for and why they were included | Pgs. 14-15 |
|  |  | (*b*) Report category boundaries when continuous variables were categorized | / |
|  |  | (*c*) If relevant, consider translating estimates of relative risk into absolute risk for a meaningful time period | Pg. 14-15 |
| Other analyses | 17 | Report other analyses done—eg analyses of subgroups and interactions, and sensitivity analyses | Pgs. 13, 15-16 |
| Discussion | | |  |
| Key results | 18 | Summarise key results with reference to study objectives | Pg. 17 |
| Limitations | 19 | Discuss limitations of the study, taking into account sources of potential bias or imprecision. Discuss both direction and magnitude of any potential bias | Pg. 24 |
| Interpretation | 20 | Give a cautious overall interpretation of results considering objectives, limitations, multiplicity of analyses, results from similar studies, and other relevant evidence | Pgs. 17-23 |
| Generalisability | 21 | Discuss the generalisability (external validity) of the study results | / |
| Other information | | |  |
| Funding | 22 | Give the source of funding and the role of the funders for the present study and, if applicable, for the original study on which the present article is based | Pg. 28 |
